# Technology for dementia care: what would good technology look like and do, from carers’ perspectives?

**DOI:** 10.1186/s12877-023-04530-9

**Published:** 2023-12-16

**Authors:** Ruth Brookman, Simon Parker, Leonard Hoon, Asuka Ono, Atsushi Fukayama, Hisashi Matsukawa, Celia B. Harris

**Affiliations:** 1 MARCS Institute for Brain, Behaviour and Development, Western Sydney University, Penrith, NSW Australia; 2 Deakin University, Geelong, VIC Australia; 3 Nippon Telegraph and Telephone (NTT), Tokyo, Japan

**Keywords:** Dementia care, Assistive technology, Unmet needs, Social engagement, Carers, Technology design ideas

## Abstract

**Background:**

The development of technology in dementia care has largely been without consultation with carers, and has primarily focused on safety, monitoring devices, and supporting activities of daily living. Further, while involving end-users in the design of technology has been recommended, this is yet to become common practice.

**Method:**

We conducted a mixed methods study with the aim of investigating carers’ values and priorities for technology development, including prior experiences, barriers to use, and what they would like technology to do. Importantly, we asked carers for their design ideas and bespoke technology solutions for future development.

**Results:**

Carers of people living with dementia (*N* = 127), including both unpaid (*n* = 102) and paid carers (*n* = 25) residing in Australia, completed an online survey. In addition, a subsample of carers (*n* = 23) participated in semi-structured interviews. Findings demonstrate that carers want technology to be person-centred, customisable, and to increase opportunities for meaningful social connection. Findings also demonstrate the ability of carers to generate creative design solutions for dementia care.

**Conclusions:**

These findings and implications will be discussed in relation to the importance of co-design with carers and engineers during the design phase of assistive technology. Also, the importance of technology to enhance, not replace, human-to-human social interactions is highlighted.

**Supplementary Information:**

The online version contains supplementary material available at 10.1186/s12877-023-04530-9.

## Introduction

People living with dementia (PLWD) are a growing population, with dementia affecting over 55 million people globally [1]. An even larger population of carers are impacted by the disease, including unpaid carers such as family and friends; as well as paid carers like health and aged care staff [2]. The common feature of different types of dementia is that they lead to the progressive loss of ability to independently perform everyday tasks such as dressing, grooming, washing, preparing a meal or making a phone call, as well as social disconnection and loneliness for both PLWD and their family carers [3]. Given the range of cognitive impacts associated with dementia, there is a great deal of focus on the potential for technology to support the independence and care of PLWD, and the quality of life of carers. Assistive technology has been described as technology devices that enable someone to complete a task that they would otherwise not be able to do or to complete it in an easier and safer manner [4]. In the context of dementia care, assistive technology has demonstrated potential to address some of the long-term care needs of PLWD and their carers [3] and can engage with a consumer-driven model that promotes ‘active ageing’ and/or improvements to participation and quality of life for PLWD [5]. For carers, benefits of assistive technology can include a reduction in the demands of caring, more opportunities for enjoyment and meaningful activities, and reduced stress [6].

### The role of assistive technology in dementia care

In recent decades, there has been a growth in research examining the role that digital technologies can play as a tool to support carers by compensating for the functional cognitive and physical decline on the PLWD’s capacities to engage in everyday life [6–8]. Emerging technology may also play a key role in addressing broader social issues arising from population ageing, including a shortage of care staff and high care costs associated with older adults living within formal residential care settings [2]. According to Blackman et al. [9], assistive technology can be categorised according to their ‘generation’, from first- and second-generation devices involving low-tech devices with some improvements such as automatic detection of hazards, to third generation devices such as complex smart-home systems, to the fourth generation of highly human-like social and service robots. That is, a wide variety of assistive technologies have been developed and researched for people with dementia, focused on a wide range of different functions and methods of operating.

There is some research evidence to support the value of assistive technology in addressing unmet needs for PLWD and their carers. For example, to mitigate the risks of isolation and the pressure on carers to meet the social needs of PLWD, robots have been employed in care settings to provide social support, companionship, reminiscence therapy, and stimulation [4, 10, 11]. Research findings suggest benefits such as mood enhancement, social engagement, and a reduction in agitation, anxiety, and loneliness [12, 13]. Also, to address carers’ safety concerns [6, 8], and the risks associated with PLWD “wandering away”, wearable monitoring devices and movement sensors have been developed [4]. In addition, assistive technology has been trialled to support the completion of ‘activities of daily living’ (ADL) such as hand and body washing, toilet assistance, dressing, food preparation, and cleaning (e.g., [14–17]). Familiar interfaces such as computer screens have been used to help orientate PLWD to the time of day, resulting in an observable reduction in late-night phone calls made by participants to carers when distressed or confused [2, 18].

The research evidence concerning the benefits of assistive technology in dementia care is inconsistent [2]. Their use with high-stakes activities such as taking medication, for example, can be problematic when the device is not 100% accurate or effective in ensuring an activity is completed [19]. Complex interfaces can also exacerbate rather than reduce PWLD’s frustration and distress levels [20, 21] and there are ethical concerns around the potential privacy invasion associated with video monitoring and tracking of PLWD [22]. There are also barriers and limitations to the use of social robots in dementia care, such as their high cost, concerns about their childish appearance, individual resistance, the inability of the PLWD to engage with a robot, and ethical concerns that robots will be used to displace human interaction [23, 24]. Further, relatively few devices have been developed to support existing and new relationships between people, such as enabling more frequent social contact between PLWD and their friends and family, which presents as an under-developed area of technology development. This highlights the need for further research to establish best practices for the routine adoption and use of assistive technology.

### Challenges to the development of assistive technology in dementia care

The development and evaluation of the efficacy of assistive technology in dementia care can be challenging, and many devices have been developed for research purposes only, not for largescale development and clinical use. General challenges include the usability and acceptability of technology for older populations, the different effects of technology on different people, and the ethical considerations associated with use with a vulnerable people group [22]. Specific barriers include the complexity of the device, a lack of familiarity with the technology, the PLWD’s difficulty remembering to use the device and/or recall where they put it, and the inability to “trouble-shoot” when something goes wrong [6]. These challenges are further exacerbated by the progressive nature of dementia. Despite recommendations to the contrary, assistive technology has not historically been adaptable to the ever-changing needs of PLWD and their carers [25]. Similarly, the use of technological devices designed for the general population (e.g., smart phones and Google Home), has been constrained by a mismatch between the complexity of the devices and the needs of PLWD and carers. Perhaps because of these challenges, technology solutions have frequently emerged from a “one-size-fits all” approach that is not consistent with person-centred care. For instance, voice-activated technological home systems require initiation on the part of the user, which may be a lost skill for those in the middle and later stages of dementia.

### Carer perspectives in the design process of assistive technology

Carers have reported limited adoption of technology due to the lack of affordability, poor installation and fit of the device into the caring environment, limited information, training, and support to use of the device [7]. Carers have also reported an increase to their sense of ‘carer burden’, whereby efforts to implement the assistive technology added to their caregiving load [7]. Identifying the unmet needs and the priorities of carers before mapping technology solutions onto those already impacted by the stressors of their caregiving role, will facilitate the development of more useful assistive technology [7]. However, a significant challenge facing the developers of assistive technology – engineers with expert technology knowledge but limited dementia experience – can be in identifying the needs of carers and adapting to the variability of these needs over time. When carer-perspectives have been sought, they detail a desire for technology that it is easy to use, reliable, offers practical support, integrates with other support such as health systems, and is customisable. Despite this knowledge, prevalence studies indicate that the uptake of technology remains low, and purchased devices are frequently abandoned and remain unused [6]. This suggests a need for more in-depth consultation with carers to identify the most effective ways to overcome barriers to the adoption and ongoing use of assistive technology in dementia care [6], and also to harvest technology design ideas from those involved in the hands-on-care of PLWD.

To the authors’ knowledge, there are no studies that have examined the carers’ lived experience and its relevance for the design of person-centred technology solutions in dementia care. This is despite survey data indicating that caregivers want to be involved in the developmental process of new technology [6]. Identifying carers’ experiences with assistive technology, the ways in which it has been useful and not useful in everyday care, as well as their vision for how technology would address this gap. This person-centred consultation with end-users would also inform the future design and development of assistive technology from the perspective of carers.

### The present study

In two iterative studies, our overall objective was to bridge the gap between unmet needs, and carer experience and perspectives in assistive technology design ideas. First, we aimed to identify carers’ perceived needs to focus assistive technology interventions and best practice in dementia care. Second, we aimed to explore carers’ experiences with assistive technology and record their design ideas for future technology to solutions to improve dementia care in both home and residential settings. These aims were addressed through conducting an online survey with paid and unpaid carers, and in-depth interviews with a subset of carers. In both data collection procedures, we adopted a person-centred approach, by including open-ended questions about what priorities and opportunities carers perceived for technology development.

Specific research questions are detailed below:

1. *Needs*: What do carers identify as the most important dementia-related needs for PLWD and themselves?
2. *Experiences:* What are carers’ day-to-day experiences – barriers and benefits – of assistive technology in dementia-care settings?
3. *Design solutions:* What would good technology look like and do from carers’ perspectives: What are carers’ design ideas for novel dementia-care technology solutions in home and residential care settings?

It is anticipated that findings will guide the development of technology solutions that enable those with dementia to live independently for longer, receive a higher quality of life, and meet needs such as autonomy and privacy, while simultaneously reducing carer burden [2].

## Methods

### Research context and approach

The study was conducted in Australia with paid and unpaid carers. Australia is a large continent with a small population, 80% of which is located primarily in five major cities. Similar to other countries, the incidence of dementia in Australia is increasing [26], and there is a demand for unpaid carers that corresponds to a shortage of paid carers [27]. Approximately one third (36%) of Australian people with dementia are living in the community, and just over a half (55%) are receiving support from paid and unpaid carers [28]. There are approximately 134,900 to 337,200 unpaid carers, almost all of whom are providing continuous care (60 or more hours per week) [28]. Carer services are not always accessible, and there are policy efforts to increase the support available to carers of PLWD [27].

The qualitative component of this research was conducted under a realism paradigm [29] which occupies the middle ground between constructivism (an individual’s experience of reality) and positivism (50), and values the use of multiple data collection procedures and analytical approaches to compare perceptions of reality. As such, the present study employed a concurrent nested and sequential explanatory study design to incorporate our mixed methods approach to data collection and analysis [30]. The mixed methods approach occurs across both iterations of the study from data collection to analysis.

**Fig. 1:**
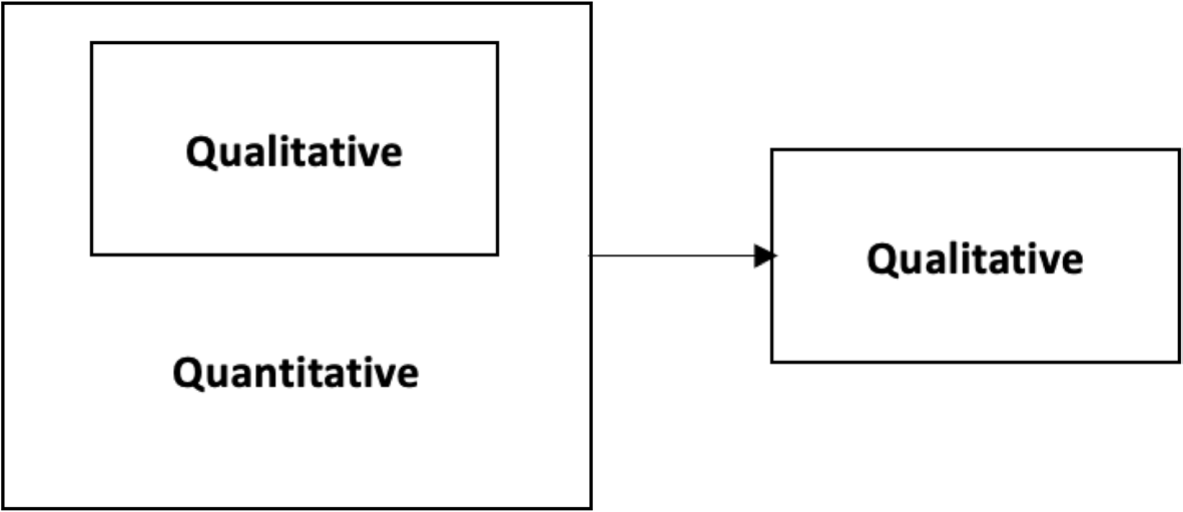
Diagram of the concurrent nested and sequential exploratory study design

The first round of data collection in the concurrent nested design consisted of an online survey in which quantitative and qualitative data were collected (see Fig. 1). The predominant data collection method was quantitative in nature. However, open-ended questions were nested within the survey, which enabled the integration of both qualitative and quantitative data in our analysis. The survey results informed a subsequent phase of data collection, which involved more detailed interviews with a subset of survey participants. The subset of interviewed participants self-selected in response to an invitation at the end of the survey. This follow-up phase adopted a sequential exploratory design and involved qualitative data obtained through semi-structured interviews. The aim of these interviews was to enable more in-depth exploration and interpretation of the findings from the surveys.

### Materials

#### Survey

The online carer survey consisted of closed- and open-ended questions designed by researchers to fit into three main sections; 1. Demographic and Background information, 2. Unmet Needs, and 3. Experiences with Technology. The survey was designed collaboratively by the research team with the goal of understanding the priorities of carers and their experiences of caring, as well as their experiences and opinions about the role of technology in supporting care.

In Sect. 1 (“Background”), participants provided demographic information (age, gender), details about their experience with dementia, their role in dementia care (e.g., spouse, nurse, etc.) and how frequently they interacted with PLWD.

In Sect. 2 (‘Unmet Needs’), participants were asked the following open-ended questions; "In your opinion, what are the three issues or difficulties that impact the most on the lives of PLWD?” and “In your opinion, what are the three issues or difficulties that impact the most on the lives of carers of PLWD?”. Participants were also asked to respond to closed-ended questions using a 5-point Likert scale (1 = ‘little or no assistance’; 5 = ‘a great deal of assistance’) to rate how much assistance was/is required for the PLWD in their care to complete activities across the following life domains: 1. leisure, 2. financial, 3. mobility and transport, 4. socialisation, 5. basics of home, food and clothing, and 6. physical health. The items for assessing needs were adopted from the Disability Assessment for Dementia Scale (DAD) [31]. For each domain, an open-ended question was also asked, “What strategies have you used to support yourself or a PLWD with these kind of activities?”.

In Sect. 3 (‘Experiences with technology’), participants were asked to use a Likert rating scale (1 = ‘never used and not interested’, 2 = ‘never used and interested’, 3 = ‘used a little but have not continued to use it’, and 4 = ‘currently use this support’) to rate how frequently they used different forms of support (e.g., medications, home modifications, and assistive technology). Open-ended questions were also employed to explore participants’ experiences regarding the benefits and barriers to using technology in dementia care. Example questions include: “What might be *helpful* about using technology in the context of living with dementia?", and “What might be *hard* about using technology in the context of living with dementia?”. In addition, respondents were invited to provide their email address and/or telephone number if they were interested in participating in an in-depth interview about their responses.

#### Carer interview

The semi-structured interview employed a set of core interview questions designed to explore the carers’ experiences with caring for PLWD, their unmet needs, experiences with technology (barriers and benefits), and design ideas for future technology. The questions were also designed to explore the strategies that carers had found successful in addressing barriers experienced by the PLWD. Example questions included: “Have you tried any strategies or tools to support your care?” and “Imagine you can invent a new technology that will support dementia care—what would it do?” (See Additional file 1).

### Procedure

The Qualtrics survey platform was used to manage data collection of the online carer survey. The survey was completed anonymously and in English. Participants were invited to access an online survey via an online link or QR code and to complete the survey at a time that was convenient to them from their home. Survey completion took approximately 30 min. An opening screen provided information about the study, and participants were informed that by continuing they were indicating their consent to participate in the study. For the analysis, we only took into consideration responses from surveys where participants had completed more than 70% resulting in a total of 127 responses. In return for their participation, participants were invited to leave an email address and be entered into a draw to win one of ten $100 AUD gift vouchers, which were drawn and issued at random.

The follow-up semi-structured interviews with carers were scheduled in September through to November 2020. Interviews were conducted remotely via Zoom to accommodate social distancing restrictions associated with the COVID-19 global pandemic, whereby university policies periodically restricted researchers from conducting conduct face-to-face interviews with vulnerable population groups such as participants over the age of 65 years, depending on the prevalence of the virus and government-implemented lockdowns. One interview was conducted face-to-face in the participants’ home, after the COVID-19 restrictions had eased. Interviews were conducted by a female interviewer, the first author (PhD/M.Clin.Psy) an associated research fellow and registered psychologist, with experience conducting interview with vulnerable populations. The interviewer did not have a prior relationship with participants, who were aware of the interviewers’ professional backgrounds and research interests. The interviewer had previous lived experience as a secondary carer of a family member with dementia, which motivated her research interests. All core interview questions were open-ended and phrased conversationally, to allow maximum flexibility in accommodating the needs of participants. The mean length of each interview was approximately 1 h (mean: 66 min; range: 44–88 min). The content of the interviews was audio and video recorded, and data files were saved for later transcription and analysis by researchers.

### Data analysis

Survey data and closed-ended questions were statistically analysed using SPSS 22.0. Participant responses to the open-ended questions were tabled in an Excel spread sheet, scanned for difference and similarity, read for each participant, and then inductively coded by the first and last authors, both female, using content analysis and a thematic analysis approach which is a method for identifying, analysing, and interpreting patterns of meaning (or ‘themes’) within and across participant responses [32].

Participant interview audio files were transformed into a written transcripts using automatic transcription software, Otter.ai. These initial transcripts were then manually checked by researchers for accuracy against the audio recording. Written transcripts of the interviews were carefully analysed by the first and last authors using thematic analysis [32]. Both authors (females) had post-graduate training and experience in qualitative research and analysis. The first author was employed as an associate research fellow and registered psychologist and had previous lived experience as a secondary family carer of a person with dementia. The lived experience motivated her research interests and may have created bias through identification with interviewees. However, this was mitigated by her clinical training and experience. The last author was employed as a senior research fellow in cognitive neuroscience. Transcripts were compared with each other as themes were identified. Participants did not provide feedback on the data. No additional software platforms were used to manage the data. Themes were not identified in advance but were derived from the data.

## Results

### Participants

Participants (*N* = 127) were recruited via online networks of Australian carers and across social media, including groups for Australian carers, professional networks of nurses and aged care workers, and a sponsored Facebook advertisement. The majority of participants identified themselves as unpaid, familial carers (*n* = 102). Of these, 18 were currently in a full-time caring role, 50 were friends or family of PLWD, 13 were previously caring but no longer, and 21 were previously friends or family of people living with dementia. Unpaid carers were most frequently the adult child (68%) of a person with dementia (i.e., caring for a parent or parent-in-law), but relationships also included spouses (14%), grandparent/grandchild (3%), aunt/niece (4%), siblings (4%), and friends (7%). Unpaid carers (90% female, 10% male) were aged on average 60 years and ranged in age from 29 to 86 years. The remaining participants were paid, formal carers (*n* = 25). Of these, 19 were currently caring and six were previously caring. Formal carers had a diverse range of roles, and included nurses, nursing home managers, support workers, pastoral carers, physiotherapists, and neuropsychologists. Formal carers (96% female, 4% male) were aged on average 54 years and ranged in age from 27 to 74 years. Formal carers supported individuals with dementia at all levels of disease progression, ranging from mild to severe.

Detailed interviews were conducted with a subset of 23 carers (unpaid *n* = 17; paid *n* = 6) who had completed the survey. The invitation to participate in the follow-up interview was placed within the body of the online survey. Interested survey respondents were invited to provide their contact details at the end of the survey if they wished to schedule a follow up interview. All participants who indicated interest and who responded to our invitation to schedule an interview time with us were interviewed.

### Most important needs for people living with dementia

#### Survey responses

**Fig. 2:**
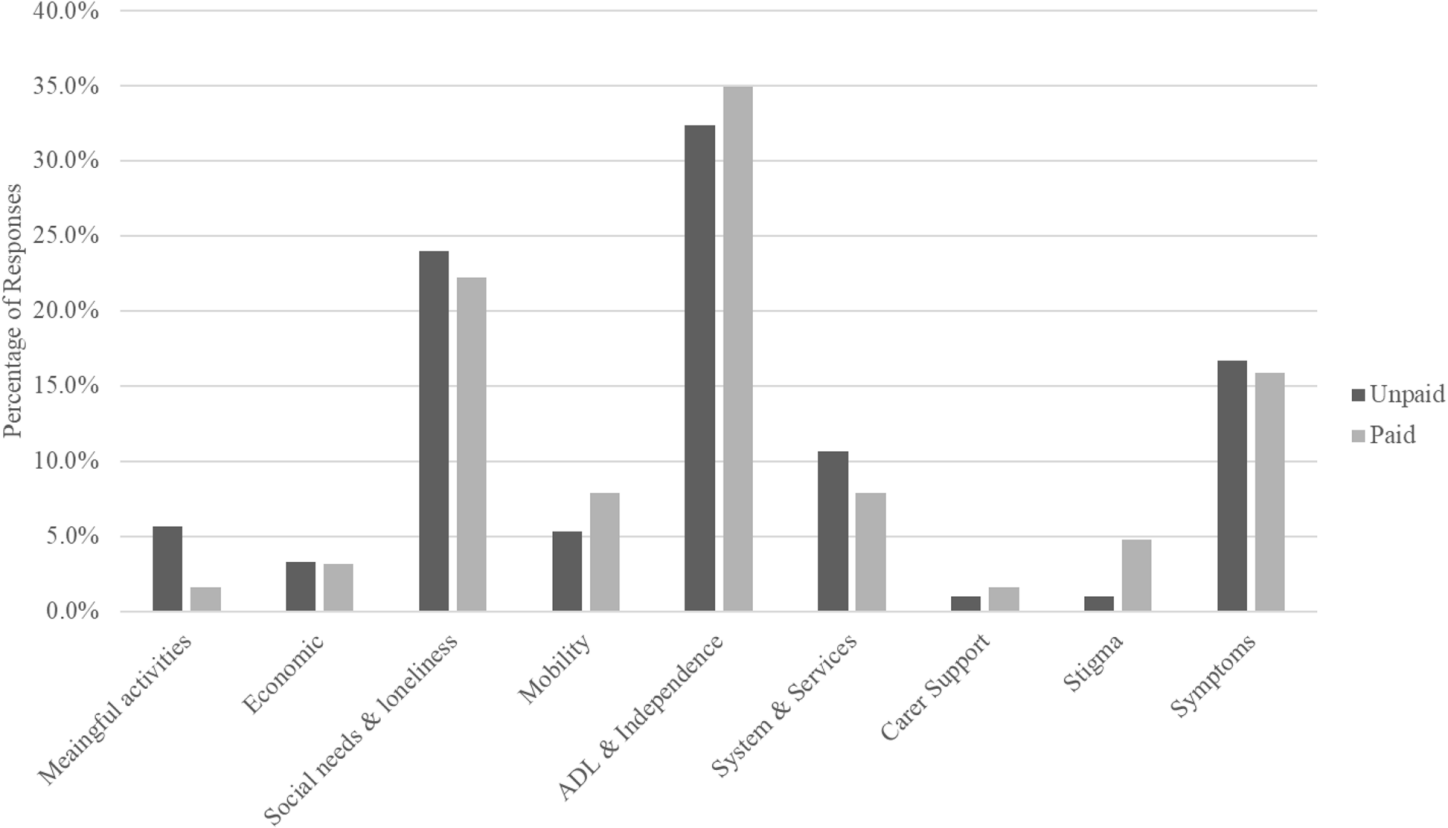
The most important needs for people living with dementia

To understand the domains where PLWD might need support from technological solutions, the survey asked carers to describe the three issues or difficulties that impacted PLWD and themselves as carers. Participants had space to provide up to three separate responses. We coded all responses to determine most common themes. Responses could be broadly clustered under 9 emergent themes or domains. The distribution of responses was virtually identical for unpaid and paid carers, and chi-square analysis indicated no differences in the distribution of responses between carer types, *X*(8) = 7.04, *p* = 0.533 (see Fig. 2).

The most common needs described by carers were associated with completing ADL and/or maintaining independence. This included general statements (e.g., “independence”; “short term memory loss”; “forgetfulness”; “difficulty following instructions”) and references to specific activities (e.g., “hygiene and feeding self”, “difficulty working out day and time”; “eating well”; “medications”; “safety at home”; “being up and down all night”).

The second most common domain for needs of PLWD related to the need for social engagement and maintaining connections. This included difficulties maintaining relationships (e.g. “inability to recognise loved ones”; “not engaging with the family”; “friends losing contact”; “need for meaningful connections with others”; “being included”), difficulties with social situations (e.g. “interacting with people”; “loss of social confidence”), challenges with communication (e.g. “stopped using hearing aids”; “unable to express feelings”; “difficulty hearing or following conversation”; “not making sense”), and feelings of isolation and loneliness (e.g. “feeling disconnected from others”; “feeling alone”).

The other domains in our analysis were mentioned less frequently, and included a need for meaningful activities (e.g., “boredom”; “not having a sense of purpose in the day”), difficulties with managing finances and decision making (e.g., “money management”; “vulnerability to scams”; “banking and bill paying”), and difficulties with mobility and transport (e.g., “can’t go out”; “forgetting where he is going”; “sitting all the time”). We also coded several responses as mentioning specific psychological symptoms of dementia (e.g., “anxiety”; “compulsive behaviour”; “confusion”; “apathy”). Several comments referenced social issues such as a need for better social services (reflected prominently in the next section on carer needs), better carer support, and the stigma surrounding ageing and cognitive impairment. Figure 2 presents the frequency of responses across domains and carer types, noting that each individual provided up to three responses.

#### Carer interviews

The interview responses aligned with the stated needs in survey responses. ADL and maintaining social engagement also arose as important areas of need for PLWD and their carers.

##### Independence

In terms of independence and managing ADL, carers spoke about the ways in which cognitive changes associated with dementia led to difficulties keeping track of daily tasks and the completion of activities, impacting on the PLWD’s ability to continue living independently.

P2. (Unpaid/family carer). I mean, he can wash the dishes if I asked him, but that's as much as he does.

P3. (Unpaid/family carer). He literally is totally dependent on mum, to take him wherever he wants to go, because he now can no longer just take the car.

P6. (Unpaid/family carer). She struggles with everything. She can't remember if she's eaten or not. She can't remember if she's taking medication or not.

P9. (Unpaid/family carer). She was trying to make something she'd made all her life. And she just said, “I tried to make your Raspberry slice and I can't do it”. You know, it was a loss ... I think the biggest thing for [mum] was that she started to feel that she was useless.

##### Social engagement and loneliness

Carers spoke about the importance of PLWD maintaining connection to their sense of identity, which can be reinforced through supporting relationships with others and social engagement. This is especially important as loneliness and isolation can be exacerbated by the loss of communication and memory skills that accompany dementia. As one paid carer noted, “They’re not just somebody living with dementia, but somebody that lived a really great life … and we need to keep that alive”. The loss of ‘memory of a relationship’ was reportedly exacerbated by geographical separation and the stay-in-place strategies associated with the COVID 19-pandemic. Also, most family carers reported that the PLWD tended to lose their memory of key family members who resided interstate, or overseas, and who were unable to physically visit the PLWD on a regular basis. This was especially the case as the disease progressed and the PLWD could no longer recognise people on the phone.

P5. (Unpaid/family carer). Social interaction, like I think that the major thing is connection … All these behaviours are being caused from loneliness, and being confused, there's no one around to say 'It's okay. They'll be here soon'.

P18. (Unpaid/family carer). You do not need any medical training to be a good dementia carer, you need a lot of relationship training.

P7. (Paid carer). In residential aged care, there is such a high task focus for staff that there is not a lot of time for people to actually interact ... It's very rushed ... I see that is a big issue.

P10. (Paid carer). I think one of the other challenges is isolation and loneliness. And another one is lacking purpose. I'd say these are the, they're the big ones ... people can become lonely, and they still have … all these ongoing emotional needs just like anyone else, to know that they belong to people, that they are loved by people. And they can get very isolated.

### Most important needs for carers

#### Survey responses

**Fig. 3:**
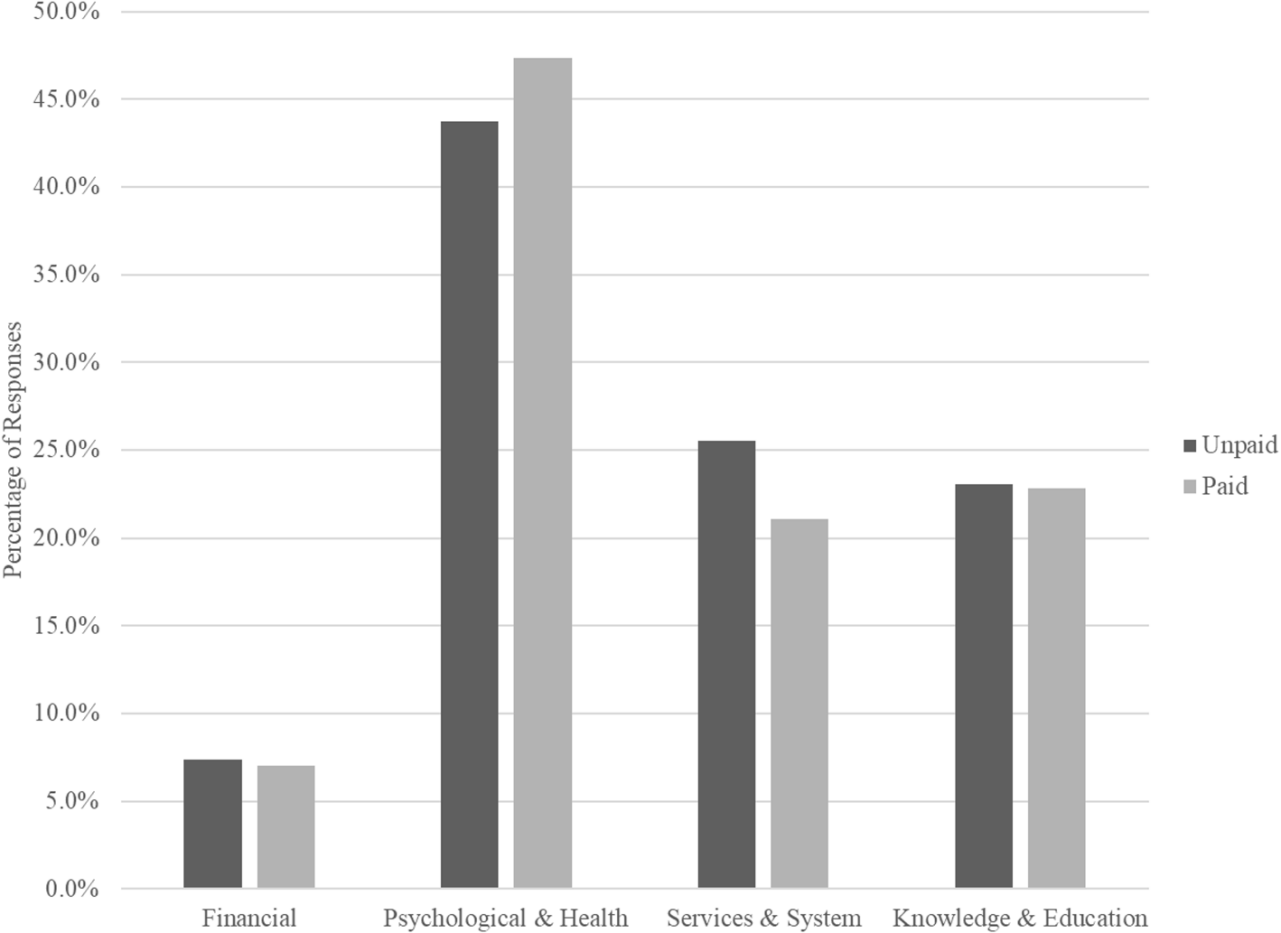
Most important needs for carers of people living with dementia

To understand the domains in which carers might most need support from technological solutions, the survey asked carers to describe, in their own words, “What are the three issues or difficulties that impact the most on the lives of dementia carers?”. Participants had space in the survey to provide up to three separate responses, that were broadly clustered under 4 emergent themes or domains. The distribution of responses was virtually identical for unpaid and paid carers, and chi-square analysis indicated no differences in the distribution of responses between carer types, *Χ*(4) = 1.86, *p* = 0.762 (see Fig. 3).

The most common domain of need reported by carers was support with their own physical or mental health. This included frequent references to being “exhausted”, “burnt out”, and “stressed”. Many mentioned difficulties with the aged care system, in terms of navigating the system, accessing support or respite, or coordinating services. Carers also mentioned having difficulty with their knowledge of dementia care, including knowing how best to interact with the person with dementia, lacking knowledge about dementia, not knowing how best to help or support the PLWD, and having difficulty determining the right balance between independence and care for the person living with dementia. Finally, carers mentioned the financial impacts of caring, particularly where they had given up paid employment in order to meet the demands of caring.

#### Carer interviews

Responses from the in-depth interviews elaborated on the potential negative impacts of the caring role on wellbeing (carer burden) and the need for carer support. There were numerous practical challenges experienced by all carers (paid and unpaid), with regards to communication, loss of independence with ADL, managing challenging behaviours, safety concerns, and time constraints in the caring role.

##### Unpaid carers

There were challenges that were unique to unpaid carers, which included issues such as social isolation in the caring role, and difficulty accessing appropriate support for themselves and the PLWD. Unpaid carers also reported distress associated with the change in their relationship to the PLWD. This was often evident when the PLWD loses their independence and/or is no longer able to perform previously held roles within their family. Many of the family carers noted that they missed the “independent and capable” PLWD and had to adjust to the increasingly “dependent” person, who eventually required 24/7 supervision and care. Many carers likened their change in role to that of becoming a parent again. As such, they valued any kind of support that enabled them to find a “circuit breaker” or “snippet of time” free from the intensive nature of their caring responsibilities.

P1. (Unpaid/family carer). But living with her was difficult because dementia, as you know, for somebody on a “sundowner” is quite difficult. So, in the afternoon, evenings, she'd often get quite distressed … And then the night times … they sleep poorly … she'd be up and down, up and down.

P9. (Unpaid/family carer). I was losing more of my identity … I had gone from being an independent, active social person to just being a ‘carer’… your will just goes down until your life is just this little pinprick. And you just have to dedicate yourself entirely to them, and you don't have a choice … you're just there.

P15. (Unpaid/family carer). Really, you just have that “circuit breaker” and it needs to be longer.

##### Paid carers

Paid carers were employed in a variety of different roles such as: nurse, nursing unit manager, pastoral care worker, aged care facility manager, support worker, hospital-based Dementia Advisor (Psychologist), and Therapeutic Engagement Specialist. During the in-depth interviews, most paid carers described their caregiving role as both rewarding (“I just feel my role is amazing”) and challenging (“… there is such a high task focus that there is not a lot of time to actually interact”). Paid carers reported a desire to spend more time with the individual PLWDs that they cared for, and to improve their capacity to provide person centred care. Those in management positions such as residential care managers or nursing unit managers also identified challenges related to staffing-patient ratios, and access to additional funding and education for their staff.

P7. (Paid carer). So, a staff member [who] is a really empathetic person … she said to me, “I can't make eye contact when I go in the room sometimes, because I'm in such a hurry … So, I often don't make eye contact, and I talk without looking” … And I said, “How do you feel about that?” And [the staff member] started to tear up and she said … “I feel dreadful, that's not why I got into the industry. And I don't know how to do it any other way.”

### Carer experiences with technology

#### Survey responses

Given the large number of assistive technologies that have been tested in previous studies, and the interest in assistive technology for a growing population of PLWD, we explored whether carers had adopted any technological solutions to meet the needs of the PLWD (or themselves) and what they found useful. We asked carers to rate different types of solutions or supports in terms of their prior use. For each solution, participants selected from the following options: “never used and not interested”, “never used but interested”, “have used but not continued”, “currently use”. We compared responses to three types of solutions: (1) care options or medical approaches; (2) everyday tools involving technology; (3) everyday tools not involving technology.

##### Unpaid carers

**Fig. 4:**
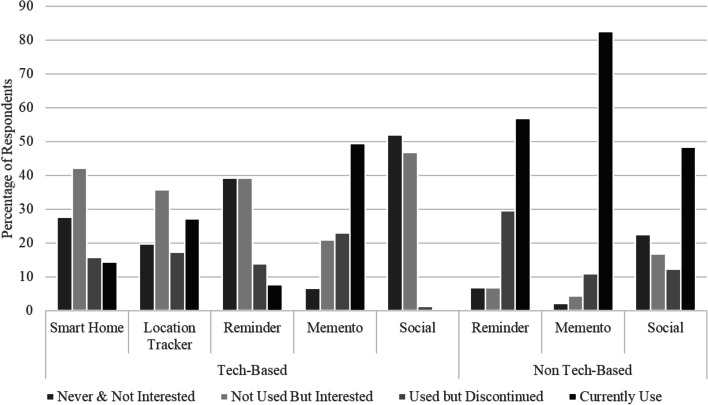
Unpaid carers’ use of technology-based and non-technology-based solutions

Ratings for unpaid carers’ use of everyday tools are presented below, showing the number of people endorsing each response option (see Fig. 4). Technological tools included smart home options such as automated lights, cameras, doorbells; location trackers or GPS-enabled wearables such as an Apple Watch or Fitbit; reminders provided by a phone or other device; memento technology such as digital photo frames or music players; and social technology such as companion robots. Most commonly participants reported that they had not previously used the technology and were divided between ‘not interested’ and ‘interested’. This reflects an ambivalence about the value of technology as many carers were sceptical regarding its value in their daily life and caring responsibilities. The one exception to this was technological mementoes such as digital photo frames or other ways of supporting reminiscing about the past, which carers reported using. The next most common technological tool being used was GPS-enabled wearables, with about a quarter of carers reporting using such devices. Strikingly, carers reported not using social robots, and were evenly split between disinterest and interest in such social support tools. Non-technological tools included reminders provided by paper tools such as calendars or shopping lists, physical mementoes such as photo albums, and physical social supports such as pets or dolls. Such non-technological tools were most frequently reported to be ‘currently used’.

##### Paid carers

**Fig. 5:**
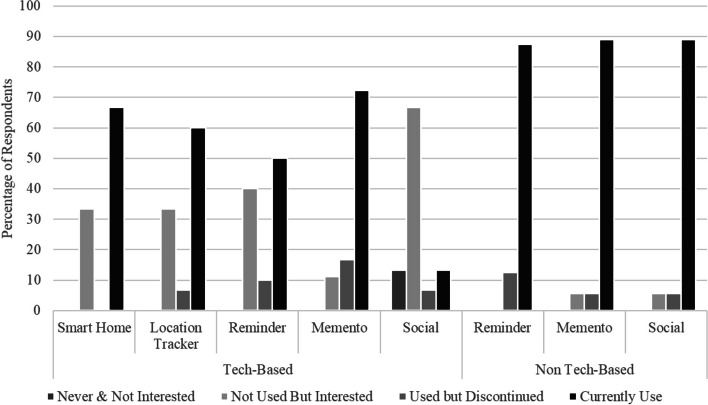
Paid carers’ use of technology-based and non-technology-based solutions

The responses of paid carers showed a sharp contrast with those of the unpaid carers. Paid carers were much more likely to report currently using a variety of tools, including both technological and non-technological (see Fig. 5). For those who did not currently use a particular tool, they were likely to express being interested in it. This pattern is especially striking for social technologies such as social robots, which were the least commonly used but of interest to most paid carers. Paid carers rarely selected the “never and not interested” option. This suggests that paid carers are engaging a range of technological and non-technological tools already and are also more open to adopting new technological solutions across device types.

### Specific examples of technology use in care practice

Follow-up interviews provided rich details about the specific ways that carers embedded technology within their practice, meeting a range of needs including addressing isolation, loneliness, and agitation and enhancing personal meaning and connection to self. This was particularly the case for paid carers who reported higher rates of incorporating technological tools into their care practice, as such the example responses below are drawn only from carers with experience using technology. Rather than using technology in a generic way, these carers (both paid and unpaid) talked about the importance of using technology in a person-centred way and lamented that “one size does not fit all” in dementia care. This was especially the case when using technology for leisure and/or to engage the PLWD in activities that were meaningful to them e.g., listening to classical (versus) popular music. Also, in a hospital setting when patients were unable to use the nurses “call bell”, because the detail was too small and complicated for them to use. The following examples demonstrate the creative, idiosyncratic, and DYI bespoke ways that carers had found to make generic technology personalised and meaningful for individuals, including focusing on personalised content or targeting particular times of day or contexts when technology use is beneficial.

P6. (Paid carer). The most amazing thing I have found is a digital day clock. It's fantastic. It comes up with the date, the day, whether it’s afternoon or morning. [For example] it would say it is 2:15 pm on Thursday afternoon. It would give the year as well. Some of them can set alarms still which is great ... I know my Lewy Body [client], her alarms are all set - timed for her medication.

P8. (Paid carer). Some families have hooked up DVD players [to the resident’ TV], others get a hard drive and load all the movies and music into the back of the TV… we've [also] got iPads and Samsung tablets that we use, and then the staff will sit down with the residents … either take them out in the garden or in their room and call their families … We've got a few that do [use it by themselves]. Not everybody because some just don't cope … they just don't understand who’s on the end of it … I've got a lady who's Italian [who] misses her family … She understands English [but] doesn't speak it as well anymore … She has a phone call every morning and then she video calls [her daughters] every evening because she “sundowns” really quite badly. And the girls will do a video call when she's getting unsettled.

P12. (Paid Carer). With some of our patients with dementia we we've got a special symbol … just a big white ‘bell’ [on a] … a big white soft button … the OTs [Occupational Therapists] got them for us. And we say to [PLWD] I'm the nurse, if you want me press this button, and… [they] can understand. Now I've got the button. If I press this button, the nurse will come.

Carers talked about different tools they had used to facilitate social interaction and reminiscing. Carers generally recognised that connecting the PLWD to their identity, and their past employment, culture, roles, and relationships was important. Tools were often non-technological, such as photos or life story books, developed deliberately by the carer to be used as a resource for sharing memories and for telling other people (e.g. paid carers) about the PLWD. Many talked about music and its ability to connect people to their past, to provide a meaningful activity and a way of engaging them. Most paid carers were open to the possibility of technology, when tailored to the individual’s needs, and recognised the potential for technology to provide tools to facilitate reminiscing, and/or engagement with PLWD, reinforcement of the PLWD’s identity, and promote relationships and connection to meaningful activities. In terms of technology for social engagement, some comments below indicated the need for multimodal stimulation to maintain the attention of the PLWD – being able to see as well as hear a person they are talking to or being able to touch as well as see photos.

P15. (Unpaid/family carer). We’ve used WhatsApp on the phone with my sister. So, because Mum’s recognition of a phone now is that she doesn’t understand what a phone is. She hears my sister’s voice, and she wants to know where she is. With WhatsApp, she could see my sister and therefore she related much better to.

P4. (Unpaid/family carer). He just likes, you know, the love of classical music [it] is probably one of the last things that to go. He came from a very musical family… Television itself distresses him, especially if it's left on accidentally between something bland, and it gets into airline disasters or something. But he has about 40 h of opera and ballet selections on USB [that I downloaded for him], which plugs directly into the television. The staff can choose something he doesn't mind watching it over and over again.

P20. (Unpaid/family carer). Dad ... he was more of a classical music person. So, he was more into Mozart and Beethoven and, and that sort of stuff. Whereas they [the staff] tend to play more the old popular music.

P10. (Paid carer). I take the iPad in everywhere, every day, because I don't know how I'm going to use it [for example] looking up places.... Google Maps, things like that. Many of the residents have an iPod too, and that's their own personalised music playlist with headphones. Because music's hugely significant for people … the family give us a list of their preferences. And then that gets put on the [PLWD’s] iPod.

In one residential care setting, subscription to a multi-media music platform was used specifically to address agitation in residents, particularly at night-time if residents were unable to sleep, and when scheduled activities were absent. This platform provided 24–7 visual footage and songs from a familiar era for the PLWD. They found this was helpful to some residents at night-time and provided a sense of company in addition to ‘calming’ visual and auditory stimuli.

P8. (Paid Carer). Silver Memories’ [is] a radio station here in [city] [that plays] continual reminiscent music that runs 24-7 with visual pictures and... it's hooked up to the facility [and] every resident’s television. So, if someone's having a hard time sleeping at night, [the PLWD or staff] can put that on … For those residents that ... go to church on Sunday, they can play ‘Hymns of Praise’ ... Because there's so many [residents] whose body clocks just do a “flip-flop” on them when they're living with dementia. So, all of a sudden, they're awake all night, and the staff during the night [need] to engage them … ‘Silver Memories’ has helped give them company - that they're not alone ... a lot of its music from the 50s and 60s ... The visuals can be just beautiful landscapes and can be anything that becomes a reminiscence thing as well.

In another residential care setting, staff had worked together to create a DYI, bespoke immersive device that included the creative use of video footage to familiar activities and settings for residents. Staff had used a Go Pro to obtain a video footage of a variety of everyday activities such as familiar car trips around the local city, bike rides through the bush, and feeding penguins. Each video footage of an activity lasts for 30 min and was played on a large screen in the recreation room. Several different “stands” were custom built for residents to physically engage while watching the virtual video footage, including a stand with a car steering wheel on it, and a stand with push bike handles that could be placed in front of residents’ chairs. The resident was then able to “drive” or go on a “bike ride through the bush” or feed the birds, thus reliving previously enjoyed activities again. The personalised content – filmed on location by staff members to match specific PLWD’s life experiences – was key to the benefit of this device, as well as the personal meaning and sense of independence provided by the simulation of driving or riding.

P8. (Paid Carer). He [staff member] put the GoPro on [his] car ... and drove from [city] to [regional town] which is about two hours away. Then [he] made me a steering wheel on the stand. I've got [the pre-recorded video] on a USB, the USB in and playing on the big screen. When I play that video on the big TV in the activity room … He [a resident] was a GP - he's ‘driving’ - he thinks he's driving up to [regional town] to see patients. We had another lady [sitting in front of the steering wheel stand] driving to wherever, she has never had a licence! And I started talking to her. She said, “Don’t talk to me, I'm driving. Can't you see that?”

Several carers noted that they had used dolls, soft toys, or basic social robots to support the PLWD, particularly noting that these tools reduced anxiety and were soothing for the PLWD. Cats/pets were the most common, with no references to more high-tech or humanoid robots. Their main function was managing the psychological symptoms of dementia rather than providing social interaction, and carers who mentioned using them noted the need to target them to the right person at the right time.

P10. (Paid Carer). I also have a robotic cat. You know, it’s got batteries, very lifelike, it meows, it purrs, it stretches, kneads its paws. And it’s been delightful, with residents who like cats. Not all residents like cats, so I don’t use it with them. And some residents know it is not real, and they still think it is amazing. And other residents think it is real. And it is very soothing.

P13. (Unpaid/family carer). Robotic cats ... I found it helped other people in the [residential] cottage, so it seems to really reassure people. And it is quite amazing really. It is life-size. It purrs and reacts to light and does all of the things a cat would do. And at Gran’s stage of dementia, they kind of think it is real. So, if I had tried that with Gran 5 years ago, she would have been really pissed off. Things have changed.

### Potential benefits of existing technology

**Fig. 6:**
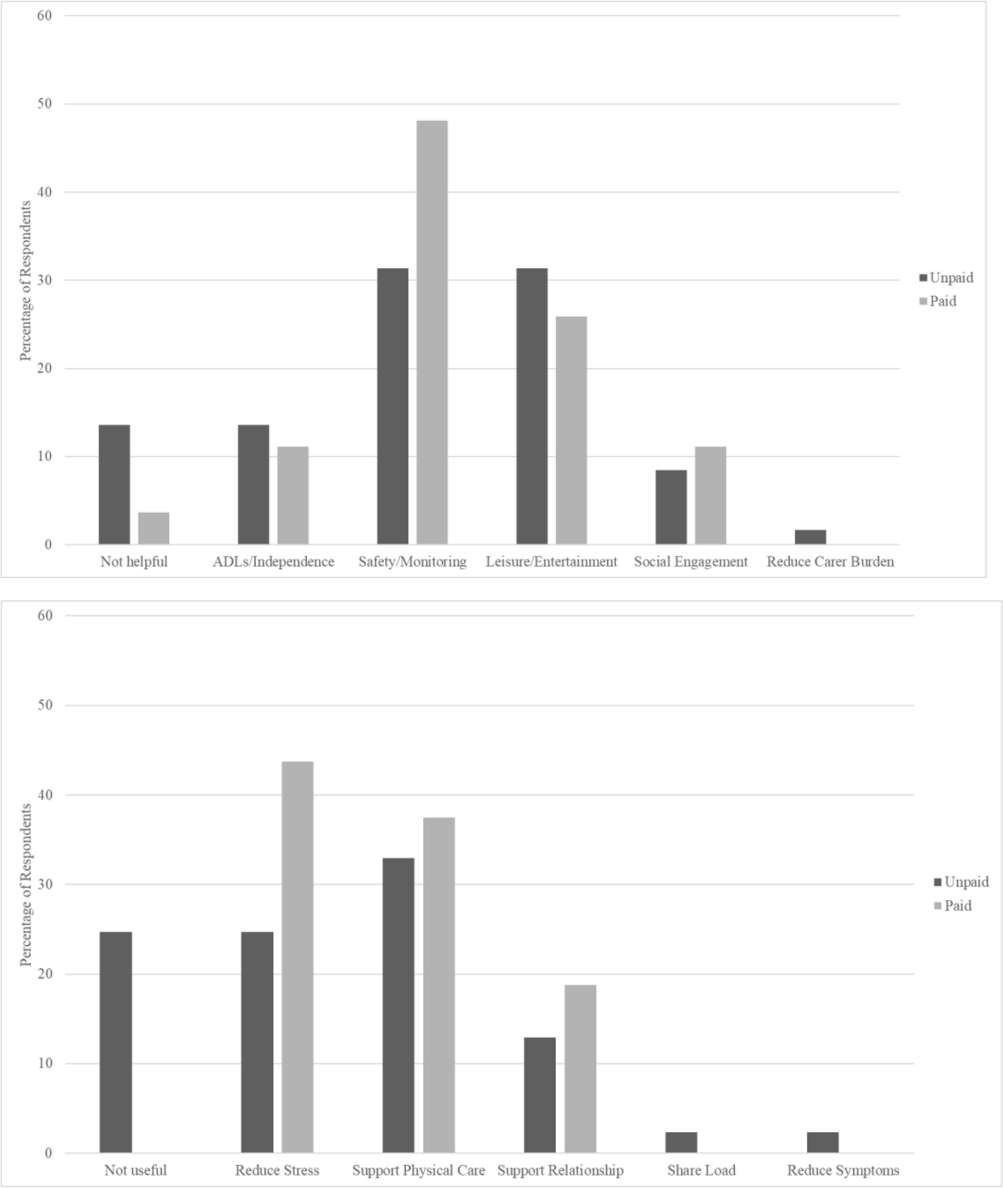
Perceived uses of existing technology for (a) people with dementia, and (b) carers of people with dementia

Given the relatively low uptake of assistive technology reported in the survey, especially by unpaid carers, we asked carers to describe what they would find existing technology useful for in the context of dementia care, both for the PLWD and for the carer, using their own words. We coded responses for the themes present, to enable us to understand what unmet needs good technology might meet (see Fig. 6). Most commonly, carers described that technology might help to support the safety of a PLWD by allowing monitoring of them in their homes. The next most common response was that technology might be useful in providing meaningful activities, supporting leisure and engagement, and reducing boredom. In terms of support for carers, responses were more likely to be that technology could reduce stress and give peace of mind, particularly through monitoring safety and alerting the carer of emergencies. Carers also described that technology could give them a break by engaging the PLWD in activities or supporting independent completion of ADL. Although the most frequent themes were similar for both unpaid and paid carers, unpaid carers (versus paid carers) were more likely to rate technology as ‘not helpful’, for the PLWD, and the carer.

### Perceived barriers to technology use

In the survey, we asked carers to describe aspects of existing technologies that make it unhelpful or present barriers to use in the context of dementia care. The most common concern or experience with technology was related to technology being “impossible to learn” and use for PLWD, who have challenges with memory and learning new information (56.67% of responses). These can be illustrated by quotes such as the following, “Mobile phone/ smartphone become impossible for him to use. Aged care homes etc. need old-fashioned phones in the rooms. So frustrating when he called and then was obviously holding the phone upside down” and “many people didn’t use technology in their lives so teaching them something new is difficult”. Carers commented on the challenges with technology interfaces, particularly their lack of familiarity to PLWD. This concern applied to all mainstream devices such as smartphones and tablets, which were reportedly too complex and too small for a PLWD to use independently, e.g. “It needs to be very simple, with one big button that can be easily identified.”

A second, related theme was the carer's observations and concern that technology use contributed to higher levels of anxiety, distress, and/or disorientation for the PLWD, meaning that introducing assistive technology could do more harm than good (21.67% of codes) e.g., “New technology causes distress and frustration—ipads/phones/ digital tv—all unfamiliar [for the PLWD].” Carers noted that PLWD have difficulties with the additional management tasks required by many mainstream devices such as turning a device on and off and charging it. They also described heightened frustration for the PLWD when they experienced difficulties “troubleshooting” or problem-solving when something unexpected happened with the technology e.g., “The issue with technology is that unless they know how to use it when there is a problem, it isn't helpful.”; “Dad would get frustrated at the alarm and inability to turn it off”. Technology is inappropriate in the context of dementia care when it makes errors, is unintuitive, or is unpredictable.

Additional concerns that were reported less commonly by carers were that PLWD could not use technology without the help of the carer (8.33% of codes) e.g., “[the PLWD] cannot use technology on their own”. Carers also expressed concern that technology could not replace human connection and that PLWD needed human interaction and not technology (5.00% of codes) e.g., “Technology doesn’t work. Mum [PLWD] responds well to being tactile, small chitchat, lots of hugs and kisses”. There was some general scepticism or concern about the push towards technology in care settings, the depersonalisation or care, and the loss of human connection especially regarding social technologies such as robots. Carers also noted that PLWD’s vision or hearing impairments made interacting with technology impossible (4.17% of codes) e.g., “Technology needs to be BIGGER if you expect [PLWD] to handle it themselves.”

### Carer design ideas: Form and function of future technology

In the follow-up interviews we asked carers to describe what they would like to see developed if they had the opportunity to ‘wave a magic wand’ and create anything that might assist in the context of dementia care. This enabled us to understand what novel technology might be most useful and valued in terms of features and function, and to suggest directions for technology development that might better align with the needs of PWLD and their carers.

#### Multi-modal technology to facilitate social engagement

Carer’s design suggestions were diverse, but most focused on facilitating social engagement that was more frequent and casual, especially when the PLWD was in residential care. Family carers wanted to be supported to interact in meaningful ways with their loved one. As such, they envisioned ways in which technology might facilitate rich multi-modal remote interactions when face-to-face interaction were not possible. This seemed particularly important for familial carers in the context of the COVID-19 restrictions and residential care lockdowns. The most frequently mentioned recommendations are illustrated by the quotes presented below.

P1. (Unpaid/family carer). If people can’t be in the room, maybe some way to ‘virtually’ do that. So, these days we're very much a fragmented society. We live all around the world … Maybe a large screen in each … room and some way that you could ‘tap into’ [it] ... So, you tap a button and you're zooming into my house. I'm in the kitchen … [the PLWD’s] in her facility, and we're just having a chat, [on a] big enough [screen] that she can see it … and it's easy to access … [as a] regular thing that you could do each day, just checking-in on things, “What you did today?”, “What did you have for breakfast?”. ... Imagine ... a big sort of interactive screen … I could see that could really work. That screen could also link in with music, which I think is really such an important thing.

P15. (Unpaid/family carer). It would be nice if the technology for the phone could work through the TV, because [PLWD] would have a bigger picture [and] they could interact much better. Because the screen on a phone is not so sharp. I mean, you could do it on a tablet … but the TV would be much, much, much better.

#### Technology to support activities at home

Family carers also made suggestions for technology that could support PLWD to complete routine personal care tasks or domestic activities at home. Suggestions ranged from a voice prompt to remind a family member to eat their lunch, to a multi-modal prompting system that could walk them through the series of steps required to brush their teeth.

P1. (Unpaid/family carer). Personal hygiene [you could] have some footage in the bathroom of someone washing their hands, because she forgets ...Whether you have someone brushing your teeth, “This is how we brush our teeth...”, “This is how we wash our hair …", “This is how we comb our hair …"

P5. (Unpaid/family carer). It would be so helpful if somebody said, “It’s lunchtime, grab your lunch out of the fridge!”. Even if you could record your own [voice]…. In our situation if I could record something that could say that, and it was timed, then that would suit my situation.

#### Capitalising on familiar interfaces that compensate for sensory losses

More generally, carers envisioned technology that harnessed familiar interfaces rather than contemporary interfaces such as smart phones and tablets which they said were too small. They therefore recommended interfaces that were large as well as familiar, such as the television.

P7. (Paid carer). I was thinking that the generation we’re talking about doesn’t really interact with computer technology, but the TV screen is a very familiar format. So, I’ve often thought we should really use the TV screen, so it is not an alien form which the computer can be … It would be great to have something with the familiarity of the TV, but I think it needs to be interactive.

P15. (Unpaid/family carer). When [Mum] sees her [sister] on the little phone. It’s too small, the screen is quite small. So, if that was the TV, I know she would be much more engaged with it.

#### Self-initiating (pre-programmed) or voice activated technology

Carers talked about the challenges of interfaces that require the PLWD to remember to use it, or to learn a complex series of steps. As a solution, several carers suggested self-initiated activation that did not require facilitation by the PLWD or a family carer. They wanted technology that required no or minimal action on the part of the PLWD. For example, a device that could be pre-programmed to dispense verbal prompts at certain times during the day, or that could be voice activated by the PLWD.

P5. (Unpaid/family carer). Definitely something that doesn’t need prompting … That can [initiate], just say, “The weather is...” or “Today is going to be hot, make sure you put on a t-shirt".

P11. (Unpaid/family carer). I suppose if she could have a screen as big as a big screen.... maybe if she could voice activate it. If I had a big screen like I’ve got here on my computer now, and she said, “What’s the weather like?” or “Can I see my grandchildren’s photos?”. That would be wonderful. That would give her a sense of contact with people ... That would give her some entertainment … Something where she could say, I want to do a crossword or a quiz or something, and you could have the clue written in big letters. She’s missing the stimulation that she has when she has people contact.

#### Technical support for carers

Finally, as a minor theme, some carers were aware of their own challenges in learning new technology and often reported feeling out of their depth when engaging with new devices or knowing what to try. They wanted access to technology support and recommendations for technology that would be appropriate for their circumstances.

P21. (Paid carer). But you need a tech support line. So, it's like, “Is there a device that can do this?”, or “How do I go about setting up a webcam at home?”, or “What are some good apps?”.

## Discussion

We aimed to understand the day-to-day lived experience of unpaid and paid carers of PLWD, including their needs and experiences with various kinds of assistive technology and their design ideas for future technology. We analysed responses from carers who completed and online survey and from carers who participated in and in-depth interviews. Results indicated that the *needs* in dementia care were widespread, with the main themes confirming previous findings by identifying the need for independence in ADL, social engagement, and management of negative psychological symptoms such as anxiety and frustration. Results from questions exploring *experiences* with assistive technology, indicated that unpaid carers reported low rates of use, and their responses reflected some scepticism about their value. Unpaid carers (versus paid carers) were more likely to rate technology as ‘not helpful’. Also, both paid and paid carers practiced the adaptation of existing technology to make it more personalised in order to better meet the needs of the people they cared for. Finally, carers’ *design solutions* demonstrated the capacity to generate creative technology ideas, with most focused-on technology facilitating social engagement that was more frequent and casual, especially when the PLWD was in residential care. They also wanted technology that was customisable, large in size, and that incorporated familiar interfaces.

All carers (paid and unpaid) emphasised the importance of person-centred approaches to dementia care, and the need to support individuals to maintain their individual identity and connection to their life story and relationships. PLWD value opportunities to engage in personally meaningful activities, to live a full life that is not defined solely by their dementia diagnosis [33].

### Technology and unmet needs

When carers answered open ended questions about the biggest issues in dementia care, the most common response related to independence and ADL, the second concerned social isolation and loneliness, and the third concerned the psychological symptoms of dementia such as anxiety and frustration. Social connection is one of the most common unmet needs in dementia care and can remain undetected [34], and PLWD want to be enable to maintain social connections with family and friends [35]. Consistent with the literature, carers in our study reported being limited in the time available to them to meet the social needs of those in their care, especially when the physical aspects of care are demanding and time consuming [36].

When carers were asked to describe what they would find existing technology useful for in the context of dementia care, the most frequently endorsed response was for safety and monitoring. Despite this, most of the solutions that carers imagined focused on social engagement and meaningful activities. These findings consistent with the literature indicating that carers adopt assistive technology in the home environment (e.g., surveillance cameras and wearable tracking devices) primarily for safety reasons to monitor the PLWD [6, 8], indicating that currently available technology may be mostly applied to this function. However, there were a diversity of needs, and a particular prevalence of unmet need relating to independence and social engagement, and these needs appear to not be well met by existing assistive technology [4]. Several common themes were identified as crucial take home messages and will be discussed below.

### Technology needs to relieve and not add to carer burden

Despite the number of unmet needs for PLWD, and the potential for technology to meet them, most unpaid carers reported that they had *not* previously used a range of assistive technology options. Carers were split evenly when given the option of being ‘interested’ or ‘not interested’ in technological solutions. Unpaid carers reported low rates of assistive technology use, and their responses reflected some scepticism about their value, while they showed much higher rates of adoption for non-technological assistance. Paid carers had higher rates of technology usage and were also interested in technology that they were not already using. This ambivalence regarding the value of technology in dementia care, may be related to carer stress and burnout. The biggest issues carers faced for themselves related to the negative impact of caring on their physical health and psychological health, using words such as “stress”, “exhaustion”, and “burnout”. Consistent with the literature, carers also reported barriers to technology use for PLWD [6, 7], particularly that technology is challenging to learn, and interfaces are unfamiliar for PLWD, contributing to anxiety and frustration rather than resolving them [20, 21]. Some carers saw potential for assistive technology to reduce the demands of caring, provide more opportunities for enjoyment and meaningful activities, and reduced stress [6]. However, to achieve this, successful technology will need a specialised user interface, reducing complexity as well as incorporating familiar ways of interacting rather than requiring the PLWD to learn a new way of interacting with a device. Technological solutions will need to integrate easily into daily life rather than adding to the load of carers. This also includes consideration of the individual needs of both unpaid and paid carers, who may not be experienced or confident with setting up and maintaining technological devices, and who are busy with many demands on their time to complete basic care tasks.

### Customisable technology

A common theme emerging from both studies was the need for technology to accommodate to the ever-changing needs of the PLWD; both across time (due to disease progression) and across the course of a day (due to environmental influences), as well as based on an individual’s life experiences, values, and preferences. This is consistent with the literature that indicates that people with early-stage dementia emphasise how important maintaining a good quality of life is for them as their disease progresses [25]. Technology solutions need to be customisable to meet the needs of PLWD as the disease progresses and symptoms fluctuate daily. It may not be realistic for PLWD to independently initiate or operate technology solutions in the later stages of the disease progression. Technology for PLWD living in residential aged care, will look very different, and may need to be operated by carers e.g., virtual activities in recreation room, or social interactions with family. The use of assistive technology needs to be person-centred and shaped around the goals and capacities of the individual.

### Adaptations of existing technology

Our findings of creative adaptation of existing technology, provides evidence that carers modify existing technology to meet their PLWD’s needs, rather than purchasing off-the-shelf products [7]. Our results extend existing research by findings that this “do-it-yourself” practice occurs in residential care settings by paid carers, in addition to home environments by unpaid carers. For example, paid staff in one residential aged care facility provided innovative examples of modifying existing technology, (e.g., Go Pro video footage), and making it interactive (e.g., steering wheel, push-bike handles), to provide bespoke multi-modal immersive interventions that were customisable and therefore person-centred e.g., “driving” the familiar route to work for a retired GP. This practice is consistent with previous research suggesting that older adults in the community tend to adopt and use technology in a contingent rather than in a systematic manner [37, 38]. Further, due to barriers associated with existing technology, and the lack of customisability, carers will act as mediators between technology and the PLWD and will tend to use available materials to adapt technology to the changing needs to the PLWD over time [7].

### Carer design solutions

Paid and unpaid carers offered creative and innovative design solutions from their lived experience, contributing unique value to the existing literature on carer perspectives. Carer design ideas included technologies that support meaningful activities (e.g., listening to music, or doing a word game), connect the PLWD to identity (e.g., reminiscing), and promote social engagement (e.g., face-to-face virtual connection on a large screen that did not involve staff mediation). Design ideas put forward by carers were rich, engaging, and multi-modal, incorporating vivid visual stimuli as well as sounds, touch, and other tools. For example, visiting the ocean “virtually” through an immersive video experience in a residential aged care setting, while also smelling the sea air and feeling the sea spray. These design ideas mapped directly onto unmet needs identified in the survey data and described functions that most carers addressed in their interviews. Carer design ideas also advocated for the use of technology that was familiar to the PLWD, such as televisions as opposed to handheld touchscreen devices. The television represents a major source of entertainment and information for PLWD [39] and could be leveraged by future technology as the platform for remote social interactions. Carers also expressed a vision for the television (versus smaller handheld devices) to be used as a platform for social interactions.

In terms of design features, carers commonly mentioned that “large” technology is necessary, and that less complexity is better. When designing solutions, the temptation to make them too complex needs to be avoided. Technology in dementia care should ideally be integrated easily into everyday life, without additional burden for the carer. For example, technology that is fixed in location would avoid issues of the PLWD misplacing and/or forgetting to use the technology. In general, carers reported that mainstream devices such as smartphones and tablets had not been useful beyond the early stages of dementia, as they are too small and complex to interact with. As alternatives, carers imagined technology that was self-initiated, and could prompt the PLWD to complete daily tasks e.g., when to eat a meal, or what to wear if it was cold. They described technology that they could be pre-programmed according to the fluctuating interests and abilities of the PLWD. Likely solutions, therefore, need to be simple, using a familiar interface, self-initiating or initiated remotely by carers, and tailorable to individuals’ needs, routines, capabilities, and interests. Very practically, this means devices that are large enough, and stimulating and multi-modal enough to engage the PLWD and avoid the issues with existing videoconferencing where the PLWD cannot follow or attend to the interaction.

Finally, in terms of the function of the solution, paid and unpaid carers emphasised the key importance of meaningful engagement, instead of the physical aspects of care. This is perhaps unsurprising given that carers often experience rupture to relationships, which can lead to social exclusion that co-occurs with the PLWD’s declining communication skills and cognitive abilities [40]. Carers wanted technology that can facilitate social engagement, especially with family. While carers reported robotic pets to have a beneficial role in residential care settings in reducing the negative symptoms of anxiety and agitation, and improving mood in [12, 13], they are not considered a solution to meet the social needs of PLWD. Unpaid carers want technology to facilitate real relationships with real people, as opposed to creating social partners that are virtual or robotic. They want technology to simple enough to be used independently by PLWD to support frequent, incidental social interactions to compliment face-to-face interactions, or to replace them when geographical separation precluded “in-person” visits.

### Implications

This is the first study to invite paid and unpaid carers to offer their creative technology design ideas to support dementia care in both home and residential care settings. Carers confirmed previous findings by articulating both their needs and limited experience with assistive technology. They also expressed a desire for technology that facilitated, not replaced human interaction. The Covid-19 lived experience of restricted access to loved ones in residential care, for both paid and unpaid carers, may have motivated the desire for user-friendly future technology that facilitates social interaction but is not dependent on staff mediation. Future technology development should consider this need as an obvious focal point for future development, particularly as Covid-19 related lockdowns and restrictions associated with outbreaks of infectious disease continue in residential aged care.

The creative adaptations and design solutions offered by both paid and unpaid carers indicate the value of co-design between front-end engineers of technology and people with close lived experience of dementia. Our findings support this collaboration as an important step in addressing the many challenges facing effective technology development. The ability of carers to adapt existing technologies and creatively generate new ones, can be leveraged to guide future technology that is customisable due to its development in consultation with end-users [6, 7, 37]. Our findings highlight carers as a valuable source of creative design ideas. As such, co-design strategies will likely ensure relevance and usability of assistive technology solutions [2]. The placement of dementia care end-users at the centre of the design process, however, is not common practice in technology research and development [37], despite the recommendation that user-perspectives should be engaged throughout the design process in dementia care [41], together with the creative design solutions offered by carer participants in this study. It is therefore recommended that carers be included in the design phase of assistive technology – in addition to the evaluation phase – to increase the usefulness and uptake of new devices in the everyday lives of PLWD and their carers.

### Limitations and future directions

It is noted that data was collected for this study during the Covid-19 pandemic and associated lockdown in Australia, during which there were stringent restrictions on the ability of families to visit loved ones in aged care. While it is not anticipated that this directly impacted on the findings of this study, the stay-in-place strategies associated with the COVID 19-pandemic may have heightened the carers’ requests for assistive technology that supports remote social interaction. Future research could examine whether the prominence of social themes remains as the restrictions on visiting aged care have eased. We also recruited a convenience sample, using online methods of contact including email and zoom interviews. This means our sample was likely more proficient with technology than average. We note that our findings regarding relatively low uptake of assistive technology are striking in the context of our digitally-literate participant cohort, and a truly representative sample recruited through other means would likely show even lower uptake of technological solutions.

## Conclusion

Our findings exploring unmet needs and technology experiences (potential barriers and benefits) of assistive technology in dementia care, are consistent with previous findings regarding carer experiences [6, 42]. However, this study extends the literature by also consulting with carers about their design ideas as end-users. The findings provide direction for future assistive technology development, including customising platforms and technology solutions so that they can meet multiple needs in different ways, as selected by the users. As such, future technology development needs to acknowledge and accommodate a broad range of ideas, depending on people’s individual circumstances, goals, and care arrangements. No single solution is going to meet all needs in all settings. The technology design phase also needs to include carers and focus on the development of several different technologies in parallel, with platforms that can be customised and adapted for different purposes.

### Supplementary Information

**Additional file 1:** Core interview questions.

## Data Availability

The dataset supporting the conclusions of this article is available in the OSF repository, https://osf.io/64zp9/
