# Supplementary material for: Technology for dementia care: what would good technology look like and do, from carers’ perspectives?
Source: BMC Geriatr. 2023 Dec 16;23:867. doi: 10.1186/s12877-023-04530-9 (PMC10725604; doi:10.1186/s12877-023-04530-9)
Supplement: Supplementary file 1 — Additional file 1: Core interview questions. [file 12877_2023_4530_MOESM1_ESM.docx]

**Additional File 1**

**Core Interview Questions**

**Participant background and experiences:**

1. If you don’t mind, could you please tell us your age or age range?
2. In what capacity do you have experience with people living with dementia (PLWD) (i.e., informal carer, formal carer, other friend or family member)
3. How long have you been interacting with a PLWD?
4. Professional carers:
5. For how long have you been working as a professional carer?
6. How long have you been caring for someone living with dementia?
7. What sort of training did you have to do to work as a professional carer?
8. Have you been provided with any specific training to look after PLWD?
9. If yes,
10. What training have you undertaken?
11. Have you found the training to be helpful?
12. If no,
13. Do you think that you need additional training to empower you to provide better care? Why?
14. According to you, do professional carers require additional training to be able to work with PLWD?

**Participant’s approach**

1. Currently, how many PLWD do you interact with/are under your care?
2. How often do you interact with them?
3. How do you feel about your dementia care?
4. Are you satisfied with the support you provide?
5. Do you feel that the person/people you care for are happy with the interactions and support from you?
6. Could you roughly tell us the stage(s) of dementia for the person/people under your care?
7. Specifically:
8. healthy (age-related cognitive decline)
9. mild dementia
10. moderate dementia
11. severe dementia
12. In your own words, could you tell us why they are classified in the category above?

**Experience with Care Challenges**

1. Can you describe a typical day? Step us through how it looks and what activities you engage in during your care.
2. What are your top 3 challenges that you encounter as someone interacting with/caring for PLWD? What activities are the hardest?
3. What are your experiences of how a PLWD can engage in the following kinds of activities? What are the barriers or ways of supporting?
4. Social and leisure activities
5. Managing money and financial activity, decision making
6. Getting around, moving and mobility
7. Basic activities of living, managing food, showering, dressing, taking medication etc.
8. Health and engaging with the health system
9. Have you tried any strategies or tools to support your care? What have you found helpful?
10. What does best practice care look like to you? What do you need to support you to engage in best practice care?
11. What do the person/people you care for want to be able to do most? What do they care about? What kinds of activities or hobbies would improve their quality of life?

**Experience with Technology**

1. Do you know of any assistive technologies for dementia care? Have you used any? Why or why not? How did you find them?
2. Imagine you can “wave a magic wand” and invent a new technology that will support dementia care. What would it be like, and what would it do?
